# Sleep Deprivation and Physiological Responses. A Case Report

**DOI:** 10.3390/jfmk4020017

**Published:** 2019-04-03

**Authors:** Marinella Coco, Andrea Buscemi, Maria Guarnera, Rosamaria La Paglia, Valentina Perciavalle, Donatella Di Corrado

**Affiliations:** 1 Department of Biomedical and Biotechnological Sciences, University of Catania, 95124 Catania, Italy; 2 Department of Research, Italian Center Studies of Osteopathy, 95124 Catania, Italy; 3 Horus Cooperative Social, 97100 Ragusa, Italy; 4 Department of Human and Social Sciences, Kore University, 94100 Enna, Italy; 5 Kore University, 94100 Enna, Italy; 6 Department of Educational Sciences, University of Catania, 95124 Catania, Italy; 7 Department of Sport Sciences, Kore University, 94100 Enna, Italy

**Keywords:** sleep deprivation, stress, exercise, daytime fatigue, physical health, cortisol

## Abstract

Background: The aim of this study was to evaluate the effects of 72-h sleep deprivation on normal daily activities (work, family, and sports), and to investigate whether sleep can be chronically reduced without dangerous consequences. Methods: The participant in this study was an adult male (age 41 years; mass 69 kg; height 173 cm). During the 72 h, data were collected every 6 h, involving a baseline (pre-deprivation). We monitored various parameters: Oxidative Stress (D-Rom and Bap test), Psychological Responses (test POMS and Measure of Global Stress), Metabolic expenditure (kJ) using a metabolic holter, EEG records, Cortisol, and Catecholamines level. Results: An interesting result was observed in the post-test phase, when a brief moment of deep sleep and total absence of a very deep sleep occurred, while an almost normal condition occurred in the pre-test sleep. Conclusion: During the 72-h sleep deprivation, no psycho-physiological stress was recorded. The participant has remained within the threshold of well-being. Only a peak was recorded during the 66th hour, but it was within the wellness threshold.

## 1. Introduction

Sleeping is a physiological process with a circadian pattern that is regulated by the suprachiasmatic nucleus, which is usually related to the light–dark alternation. This alternation determines specific behavioral habits: In general, daily and work activities are performed during the light hours, while the dark hours are dedicated to sleeping.

Several studies have addressed this topic, describing what happens during the hours of sleep. A night of sleep can be divided into phases, which are macroscopically divided into non-REM phase and REM phase [1]. The former is represented by a moment of rest and refreshment; the latter, which is typical of sleep, is characterized by vivid dreams and, among other things, it is essential for the learning processes [2].

The effects of total sleep deprivation on human beings are well documented: Evidence suggests that the consequences of sleep deprivation lead to significant physical and behavioral modifications [3,4,5,6,7,8,9,10,11,12,13]. Sleep deprivation has been shown to influence physiological and psychological functioning, having a negative effect on cognitive and psychomotor performance and mood state, partially due to decreases in creatine levels in the brain. While exercise tolerance is clearly regulated by autonomic and endocrine functions, several studies have reported no changes in exercise tolerance except for time to exhaustion under sleep deprivation. These findings suggest that the psychological effects of acute sleep loss may contribute to decreased tolerance of prolonged heavy exercise [14,15,16]. Martin [17] showed that the effect of sleep loss depends on the nature of the motor task. For example, some studies suggest that exercise is not adversely affected by sleep deprivation [18,19,20]. One determinate factor is the length of sleep deprivation. VanHelder and Radomski [21] concluded that sleep deprivation up to 72 h does not affect muscle strength or reaction but does decrease time to exhaustion. Other researchers, however, have reported performance decrements at sleep loss durations of less than 45 h [22]. However, conflicting findings mean that the extent, influence, and mechanisms of sleep loss affecting exercise performance remain uncertain. For instance, research indicates some maximal physical efforts and gross motor performances can be maintained [23].

Extreme sports are part of a particular type of sport, where those who approach this practice feel more than others the need to go “beyond the limit”, to try to experience life in a different way, “to be alive”. This search for the limit is not made to challenge life but, to the contrary, to live better, because we perceive our body and our life body in a stronger way if we experience a condition of hyperactivity [24,25,26,27,28,29,30], when our safety could be compromised, or when there are no more physical certainties (such as balance, orientation, etc.) [31,32,33,34,35].

The objective of this research was to assess whether 72 h of abstention from the regenerative rest that only the sleep phase can give, accompanied by a normal daily activity, work, family, and sports, can provide, by monitoring of various parameters, interesting information on the processes that are triggered during an effort or a prolonged stress.

## 2. Materials and Methods

An ultra-endurance walker who broke the “Longest Marathon Nordic Walking” Guinness World Record, and who decided to know his body in depth, feeling more than others the need to go “beyond the limit”, carried out this research [36].

The volunteer participant in this study was an adult male (age 41 years; mass 69 kg; height 173 cm), with normal sleep habits involving 8 h sleep per night. He was in good health, neither taking drugs nor reporting any sleep disorders. His training schedule consisted of 10 h per week distributed in outdoor and indoor sport sessions. Four training sessions per week were carried out in the gym for resistance exercise and three sessions were devoted to walking Nordic technique, a form of fast walking with two poles.

The procedures were conducted in accordance with the Declaration of Helsinki, and were performed with university human ethics committee approval. The participant was provided with a full explanation of the protocols prior to commencement of the study, and he gave his written informed consent for all tests. During the 72 h sleep deprivation the participant was in the company of supervisors participating in the experiment. He spent the following day on his usual activities (work and daily routines). Killgore et al. [37] showed that caffeine and glucose seems to be a good countermeasure for neutralizing the effects of sleep deprivation on performance and on some indices of attention. For this reason, the participant was requested not to consume kiwi, avocado, bananas, coffee, tea, chocolate, dried fruit, and sweets 24 h prior to the testing.

The parameters that were monitored during the 72 h of sleep deprivation were:

(a) Oxidative stress

The reactive oxygen metabolites (d-ROMs and BAP-test) assessment [38] was used to measure the oxidative stress. In particular, this test measures the ability “oxidant” of a plasma sample with regard to a specific substance (amine-reactive aromatic) used as an indicator (chromogen). The phenomenon is associated with the gradual and progressive color change towards the pink of the reaction mixture (plasma + chromogen), which is initially colorless. The color change is measured by a special device (photometer), which converts the oxidizing capacity so determined into a “number”.

(b) Measurement of energy expenditure

Metabolic expenditure (kJ) was monitored for the whole event by means of a metabolic holter (SenseWear Armband Pro, Bodymedia, Pittsburgh, PA, USA) [39].

(c) Heart rate

A wearable electronic device was used to assess the electrical and muscular functions of the heart [40]. An electrocardiogram (ECG) was performed at the beginning and at the end of sleep deprivation.

(d) Salivary cortisol assay

Salivary cortisol and catecholamine levels (epinephrine and norepinephrine) were measured by using blood sampling. An hour after the beginning (7:00 h) and at the end of the experiment, a 5-mL venous blood sample was taken by venipuncture from a forearm vein. Venipuncture was done to obtain the blood specimen from an antecubital vein. The blood was collected in two 3.5 mL Vacutainers™. Plasma was separated by centrifugation at 800× *g* for 20 min, and the supernatant (serum) was transferred into a polypropylene tube, which was then stored at −80 °C until analysis.

(e) Psychological assessment

- An abbreviated 30-item version of the Profile of Mood States (POMS) was used for measuring mood [41]. Respondents must complete the POMS questionnaire by rating each item on a 5-point Likert scale, ranging from ‘Not at all’ to ‘Extremely’. Internal consistency is extremely high (*r* = 0.90). Items form six separate subscales: Tension-anxiety (T), depression–dejection (D), anger–hostility (A), vigor–activity (V), fatigue–inertia (F) and confusion–bewilderment (C). The subscale scores may be combined to form an overall measure of effect that is labeled as total mood disturbance (Global score).
- The Psychological Stress Measure (PSM) was used to measure a global index of the state of psychological stress [42]. The PSM is a 49-item self-report paper and pencil questionnaire. Internal consistency is approximately 0.95. The subject was asked to answer questions about his/her psychological stress condition using a 4-point scale (very much = 4, much = 3, little = 2, none = 1).

## 3. Results

The parameters were measured 13 times: The first measurement was performed the day before the abstention from sleep considered baseline (T0); the T1 measurement was performed at 07:30 before the start of sleep abstention; the subsequent measurements were performed at intervals of six hours until its conclusion (T12).

The night before the abstention of sleep a metabolic holter was applied in order to check the presence of significant changes to be recorded and due to anxiety before the beginning of the test. During the nights of abstention from sleep, the subject undertook to carry out a small DIY project. Heart rate during the experiment did not differ from the baseline, ranging between 130 and 160 bpm.

**Figure 1:**
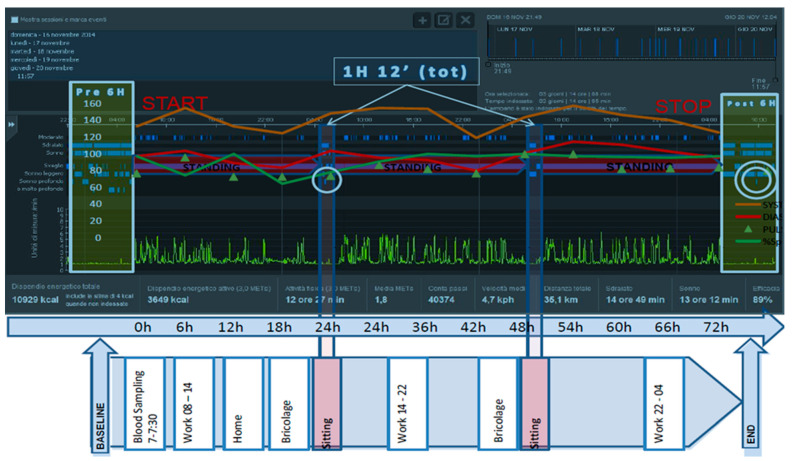
Total data measured during the 72 h of sleep abstention.

In Figure 1, it is possible to observe all the data measured during the 72 h of sleep abstention. The results obtained are within the physiological values, despite the presence of oscillations.

In Figure 1, see the white-green box, we observe the values obtained by the subject during sleep before the test and after, both 6 h in duration. The interesting element is observed in the post-test phase, in which we note a brief moment of deep sleep and total absence of a very deep sleep (double white circle), while an almost normal condition occurred in the pre-test sleep.

Concerning energy expenditure, it can be observed in Figure 1, see the red–purple bands, that during the phase in which the subject did not sleep for a total of 70 h 48 min, the daytime and nighttime activities are aligned (in yellow sequence). Moreover, the energy expenditure is 3649 Kcal, at 3 MET’s, in the active phase lasting 12 h 27 min, and 10,929 Kcal in total.

In the figure, see the box in the blue-pink column, there are two moments, of about 36 min each, where the subject reported that he sat in a chair, never lying, being at total muscle rest (flat yellow trace); primarily between the hours 5:00–7:00 am, where there is about 24 h of sleep abstention, and where a glare of deep sleep occurred (circled in white).

It should be noted that the amplitude of the deep sleep flash is very minimal compared to the overall window of the “sitting” condition. We also report what was reported by the subject during the last phase of abstention, precisely from 03:00, “I perceive as if time has stopped and I am waiting with great determination for the return of daylight in order to feel physically in tone and vigorous again”.

Table 1 shows the values obtained in the Profile of Mood States (POMS) and the Psychological Stress Measure (PSM). There is a slight modification with a peak at T11 in the PSM values, but it remains inside the wellness threshold.

Table 2 shows how the values obtained at the reactive oxygen metabolites (d-ROMs and BAP test) assessment are placed at both T0 and T12 below the minimum regulatory value. The values obtained from the measurement of catecholamines and cortisol (Table 2) are covered within the reference normative values, namely catecholamines 115.5 at T0 to move to 137 to T12 while the cortisol is 13.7 to 15.7 at T0 to switch to T12, even if there are slight increases in what is not significant.

## 4. Discussion

Sleeping is essential for every individual and the quality of sleep is a cornerstone of human physiology. In fact, in the last decades, there have been an increasing number of works on this topic, and much scientific evidence has shown that sleep disorders, such as insomnia and extremes of sleep [43,44,45], are able to act negatively on immune defense, increasing the risk of inflammation and contributing to all-cause mortality [46,47]. Though inadequate sleep has been associated with impaired cognitive and emotional function, in this study, no major psychological and physiological changes were observed. In fact, these parameters remain in a normal range.

It is well known that an alteration of the inflammatory mechanisms is able to increase the risk of a wide spectrum of medical conditions. The possible relationship between quality of sleep and physical activity is still under discussion. According to the results obtained by Semplonius and Willoughby [48] it seems that physical activity allows you to sleep better or even less frequently. The research group of Kirschen recently noted that the subjects who seem to be more susceptible to the manipulation of sleep are those athletes who play sports that require speed, tactics, strategy and technical skills [49,50,51,52].

## 5. Conclusions

In conclusion, the participant, a sportsman of endurance, chose to abstain from sleep for 72 h, in order to assess if and to what extent his mood changed. The physiological and psychological parameters remained within the threshold of wellbeing for the entire duration of the test. Therefore, we argue that in an endurance athlete the results obtained by Semplonius and Willoughby [39] are confirmed. It is probable that a person who has practiced sport for many years has probably obtained the benefits induced by sport on the quality of sleep, so less frequent sleep fails to affect their mental and physical well-being.

## Figures and Tables

**Table 1:** Profile of Mood States (POMS) and Psychological Stress Measure (PSM) total scores.

	PSM (Stress Measure)	Tension	Depression	Anger	Vigor	Fatigue	Confusion
Basal	66	41	51	43	53	39	37
T0	68	44	46	40	41	39	41
T1 (6 h)	66	46	42	41	38	41	41
T2 (12 h)	69	43	43	41	36	41	43
T3 (18 h)	60	41	42	40	36	39	41
T4 (24 h)	68	41	43	45	28	39	50
T5 (30 h)	66	41	42	40	38	39	41
T6 (36 h)	67	41	43	40	38	39	37
T7 (42 h)	62	41	42	40	33	41	43
T8 (48 h)	70	39	42	40	29	41	43
T9 (54 h)	68	43	43	43	33	39	41
T10 (60 h)	63	43	45	40	48	41	37
T11 (66 h)	73	41	42	41	28	41	41
T12 (72 h)	63	41	43	40	28	41	41

**Table 2:** Values T0 and T2 catecholamines, cortisol, reactive oxygen metabolites (d-ROMs) and Biological Antioxidant Potential (BAP) (test).

	T0	T12	Normal Ranges
Catecholamines	115.5	137	(90–720)
Cortisol	13.7	15.7	(3.7–19.4)
d-ROMs	190	190	(250–300)
BAP (test)	2690	2690	(>2200)

d-ROMs: reactive oxygen metabolites; BAP: Biological Antioxidant Potential.

## References

[1] Terzano M.G., Parrino L., Spaggiari M.C. (1988). The cyclic alternating pattern sequences in the dynamic organization of sleep. Electroencephalogr. Clin. Neurophysiol..

[2] Spiegel K., Leproult R., Van Cauter E. (2003). Impact of sleep debt on physiological rhythms. Rev. Neurol..

[3] Berger R.J., Oswald I. (1962). Effects of sleep deprivation on behaviour, subsequent sleep, and dreaming. J. Ment. Sci..

[4] Webb W.B., Agnew H.W. (1971). Stage 4 sleep: Influence of time course variables. Science.

[5] Williams H.L., Hammack J.T., Daly R.L., Dement W.C., Lubin A. (1964). Responses to auditory stimulation, sleep loss and the EEG stages of sleep. Electroencephalogr. Clin. Neurophysiol..

[6] Moses J.M., Johnson L.C., Naitoh P., Lubin A. (1975). Sleep stage deprivation and total sleep loss: Effects on sleep behavior. Psychophysiology.

[7] Nakazawa Y., Kotorii M., Oshima M., Kotorii T., Hasuzawa H. (1978). Changes in sleep pattern after sleep deprivation. Folia Psychiatr. Neurol. Jap..

[8] Reite M.L., Rhodes J.M., Kavan E., Adey W.R. (1965). Normal sleep patterns in macaque monkey. Arch. Neurol..

[9] Crowley T.J., Kripke D.F., Haiberg F., Pegram G.V., Schildkraut J.J. (1972). Circadian rhythms of Macaca mulatta: Sleep, EEG, body and eye movement, and temperature. Primates.

[10] Ursin R. (1971). Differential effect of sleep deprivation on the two slow wave sleep stages in the cat. Acta Physiol. Scand..

[11] Pappenheimer J.R., Koski G., Fencl V., Karnovsky M.L., Krueger J. (1975). Extraction of sleep-promoting factor S from cerebrospinal fluid and from brains of sleep-deprived animals. J. Neurophysiol..

[12] Takahashi Y., Ebihara S., Nakamura Y., Takahashi K. (1978). Temporal distributions of delta wave sleep and REM sleep during recovery sleep after 12-h forced wakefulness in dogs; similarity to human sleep. Neurosci. Lett..

[13] Borbly A.A., Tobler I. (1980). The search for an endogenous “sleep-substance”. Trends Pharmacol. Sci..

[14] Jennings J.R., Monk T.H., van der Molen M.W. (2003). Sleep deprivation influences some but not all processes of supervisory attention. Psychol. Sci..

[15] Vaara J.P., Oksanen H., Kyröläinen H., Virmavirta M., Koski H., Finni T. (2018). 60-Hour Sleep Deprivation Affects Submaximal but Not Maximal Physical Performance. Front. Physiol..

[16] Bruce J.M. (1981). Effect of sleep deprivation on tolerance of prolonged exercise. Eur. J. Appl. Physiol. Occup. Physiol..

[17] Martin B.J. (1986). Sleep deprivation and exercise. Exerc. Sport Sci. Rev..

[18] Martin B.J., Gaddis G.M. (1981). Exercise after sleep deprivation. Med. Sci. Sports Exerc..

[19] Reilly T., Deykin T. (1983). Effects of partial sleep loss on subjective states, psychomotor and physical performance tests. J. Hum. Mov. Stud..

[20] Samuels C. (2008). Sleep, recovery, and performance: The new frontier in high-performance athletics. Neurol. Clin..

[21] VanHelder T., Radomski M.W. (1989). Sleep deprivation and the effect on exercise performance. Sports Med..

[22] Babkoff H., Genser S.G., Sing H.C., Thorne D.R., Hegge F.W. (1985). The effects of progressive sleep loss on a lexical decision task: Response lapses and response accuracy. Behav. Res. Methods Instrum. Comput..

[23] Fullagar H.H.K., Skorski S., Duffield R., Hammes D., Coutts A.J., Meyer T. (2015). Sleep and Athletic Performance: The Effects of Sleep Loss on Exercise Performance, and Physiological and Cognitive Responses to Exercise. Sports Med..

[24] Petralia M.C., Perciavalle V., Basile M.S., Alagona G., Monaca A., Buscemi A., Coco M. (2018). The rise of lactic acid, from a pharmacist’s laboratory to entry into the central nervous system. Sport Sci. Health.

[25] Coco M., Platania S., Castellano S., Sagone E., Ramaci T., Petralia M.C., Agati M., Massimino S., Di Corrado D., Guarnera M. (2018). Memory, personality and blood lactate during a judo competition. Sport Sci. Health.

[26] Coco M. (2017). The brain behaves as a muscle?. Neurol. Sci..

[27] Perciavalle V., Blandini M., Fecarotta P., Buscemi A., Di Corrado D., Bertolo L., Fichera F., Coco M. (2017). The role of deep breathing on stress. Neurol. Sci..

[28] Perciavalle V., Marchetta N.S., Giustiniani S., Borbone C., Perciavalle V., Petralia M.C., Buscemi A., Coco M. (2016). Attentive processes, blood lactate and CrossFit^®^. Phys. Sportsmed..

[29] Perciavalle V., Alagona G., De Maria G., Rapisarda G., Costanzo E., Perciavalle V., Coco M. (2015). Somatosensory evoked potentials and blood lactate levels. Neurol. Sci..

[30] Coco M., Alagona G., De Maria G., Rapisarda G., Costanzo E., Perciavalle V., Perciavalle V. (2015). Relationship of high blood lactate levels with latency of visual-evoked potentials. Neurol. Sci..

[31] Coco M., Fiore A.S., Perciavalle V., Maci T., Petralia M.C., Perciavalle V. (2015). Stress exposure and postural control in young females. Mol. Med. Rep..

[32] Coco M., Alagona G., Perciavalle V., Cavallari P., Caronni A., Perciavalle V. (2014). Changes in cortical excitability and blood lactate after a fatiguing hand-grip exercise. Somatosens. Mot. Res..

[33] Perciavalle V., Di Corrado D., Scuto C., Perciavalle V., Coco M. (2014). Attention and blood lactate levels in equestrians performing show jumping. Percept. Mot. Skills.

[34] Perciavalle V., Di Corrado D., Scuto C., Perciavalle V., Coco M. (2014). Anthropometrics related to the performance of a sample of male swimmers. Percept. Mot. Skills.

[35] Coco M., Di Corrado D., Calogero R.A., Perciavalle Va., Maci T., Perciavalle V. (2009). Attentional processes and blood lactate levels. Brain Res..

[36] Pedrinolla A., Li Volti G., Galvano F., Schena F., Perciavalle V., Di Corrado D. (2018). Bioenergetics and psychological profile of an ultra endurance walker. J. Sports Med. Phys. Fit..

[37] Killgore W.D.S., Kahn-Greene E.T., Killgore D.B., Kamimori G.H., Balkin T.J. (2007). Effects of acute caffeine withdrawal on Short Category Test performance in sleep-deprived individuals. Percept. Mot. Skills.

[38] Costantini D. (2016). Oxidative stress ecology and the d-ROMs test: Facts, misfacts and an appraisal of a decade’s work. Behav. Ecol. Sociobiol..

[39] Bhammar D.M., Sawyer B.J., Tucker W.J., Lee J.M., Gaesser G.A. (2016). Validity of SenseWear^®^ Armband v5.2 and v2.2 for estimating energy expenditure. J. Sports Sci..

[40] Hernández-Padilla J.M., Granero-Molina J., Márquez-Hernández V.V., Suthers F., López-Entrambasaguas O.M., Fernández-Sola C. (2017). Design and validation of a three-instrument toolkit for the assessment of competence in electrocardiogram rhythm recognition. Eur. J. Cardiovasc. Nurs..

[41] McNair D.M., Lorr M., Droppleman L.M. (1992). Profile of Mood States Manual.

[42] Di Nuovo S., Rispoli L. (2000). Misurare lo Stress.

[43] Dew M.A., Hoch C.C., Buysse D.J., Monk T.H., Begley A.E., Houck P.R., Hall M., Kupfer D.J., Reynolds C.F. (2003). Healthy older adults’ sleep predicts all-cause mortality at 4 to 19 years of follow-up. Psychosom. Med..

[44] Kripke D.F., Garfinkel L., Wingard D.L., Klauber M.R., Marler M.R. (2002). Mortality associated with sleep duration and insomnia. Arch. Gen. Psychiatry.

[45] Mallon L., Broman J.E., Hetta J. (2002). Sleep complaints predict coronary artery disease mortality in males: A 12-year follow-up study of a middle-aged Swedish population. J. Intern. Med..

[46] Vgontzas A.N., Fernandez-Mendoza J., Liao D., Bixler E.O. (2013). Insomnia with objective short sleep duration: The most biologically severe phenotype of the disorder. Sleep Med. Rev..

[47] Irwin M.I., Olmstead R., Carroll J.E. (2016). Sleep Disturbance, Sleep Duration, and Inflammation: A Systematic Review and Meta-Analysis of Cohort Studies and Experimental Sleep Deprivation. Biol. Psychiatry.

[48] Semplonius T., Willoughby T. (2018). Long-term links between physical activity and sleep quality. Med. Sci. Sports Exerc..

[49] Coco M., Perciavalle V., Cavallari P., Perciavalle V. (2016). Effects of an Exhaustive Exercise on Motor Skill Learning and on the Excitability of Primary Motor Cortex and Supplementary Motor Area. Medicine.

[50] Perciavalle Va., Maci T., Perciavalle Vi., Massimino S., Coco M. (2015). Working memory and blood lactate levels. Neurol. Sci..

[51] Coco M., Di Corrado D., Ramaci T., Di Nuovo S., Perciavalle Vi., Puglisi A., Cavallari P., Bellomo M., Buscemi A. (2019). Role of lactic acid on cognitive functions. Phys. Sportsmed..

[52] Kirschen G.W., Jones J.J., Hale L. (2018). The Impact of Sleep Duration on Performance among Competitive Athletes: A Systematic Literature Review. Clin. J. Sport Med..

